# The generation of induced pluripotent stem cells for macular degeneration as a drug screening platform: identification of curcumin as a protective agent for retinal pigment epithelial cells against oxidative stress

**DOI:** 10.3389/fnagi.2014.00191

**Published:** 2014-08-01

**Authors:** Yun-Ching Chang, Wei-Chao Chang, Kuo-Hsuan Hung, Der-Ming Yang, Yung-Hsin Cheng, Yi-Wen Liao, Lin-Chung Woung, Ching-Yao Tsai, Chih-Chien Hsu, Tai-Chi Lin, Jorn-Hon Liu, Shih-Hwa Chiou, Chi-Hsien Peng, Shih-Jen Chen

**Affiliations:** ^1^Department of Medical Research and Education, Taipei Veterans General HospitalTaipei, Taiwan; ^2^School of Medicine, Institute of Pharmacology, National Yang-Ming UniversityTaipei, Taiwan; ^3^Department of Ophthalmology, Taipei Veterans General HospitalTaipei, Taiwan; ^4^Graduate Institute of Cancer Biology, China Medical UniversityTaichung, Taiwan; ^5^Center for Molecular Medicine, China Medical University HospitalTaichung, Taiwan; ^6^Division of Ophthalmology, National Yang-Ming University HospitalI-Lan, Taiwan; ^7^School of Medicine, Institute of Clinical Medicine, National Yang-Ming UniversityTaipei, Taiwan; ^8^Department of Ophthalmology, Taipei City HospitalTaipei, Taiwan; ^9^Department of Ophthalmology, Cheng-Hsin HospitalTaipei, Taiwan; ^10^Department of Ophthalmology, Shin Kong Wu Ho-Su Memorial Hospital and Fu Jen Catholic UniversityTaipei, Taiwan

**Keywords:** oxidative stress, age-related macular degeneration, patient-specific induced pluripotent stem cells, retinal pigment epithelial, antioxidant, curcumin

## Abstract

Age-related macular degeneration (AMD) is one retinal aging process that may lead to irreversible vision loss in the elderly. Its pathogenesis remains unclear, but oxidative stress inducing retinal pigment epithelial (RPE) cells damage is perhaps responsible for the aging sequence of retina and may play an important role in macular degeneration. In this study, we have reprogrammed T cells from patients with dry type AMD into induced pluripotent stem cells (iPSCs) via integration-free episomal vectors and differentiated them into RPE cells that were used as an expandable platform for investigating pathogenesis of the AMD and *in-vitro* drug screening. These patient-derived RPEs with the AMD-associated background (AMD-RPEs) exhibited reduced antioxidant ability, compared with normal RPE cells. Among several screened candidate drugs, curcumin caused most significant reduction of ROS in AMD-RPEs. Pre-treatment of curcumin protected these AMD-RPEs from H_2_O_2_-induced cell death and also increased the cytoprotective effect against the oxidative stress of H_2_O_2_ through the reduction of ROS levels. In addition, curcumin with its versatile activities modulated the expression of many oxidative stress-regulating genes such as PDGF, VEGF, IGFBP-2, HO1, SOD2, and GPX1. Our findings indicated that the RPE cells derived from AMD patients have decreased antioxidative defense, making RPE cells more susceptible to oxidative damage and thereby leading to AMD formation. Curcumin represented an ideal drug that can effectively restore the neuronal functions in AMD patient-derived RPE cells, rendering this drug an effective option for macular degeneration therapy and an agent against aging-associated oxidative stress.

## Introduction

Retinal degeneration is one of the common sequelae of aging, among which age-related macular degeneration (AMD) has become a leading cause of irreversible blindness worldwide (Friedman et al., 2004; Song and Dunaief, 2013). According to clinical presentation and ancillary examinations, AMD is further subdivided into wet and dry type (Landa et al., 2011). The slowly progressing and more frequent dry AMD is characterized by the presence of an irregular area of depigmentation as a result of the loss of retinal pigment epithelial (RPE) cells, and causes gradual geographic atrophy of the retina (Bowes Rickman et al., 2013). The well-established risk factors associated with AMD are advanced age, diet intake, cigarette smoking, and racial variety (Nowak, 2006; Tokarz et al., 2013). The complement factor H gene (*CFH*) and 10q26 containing age-related maculopathy susceptibility protein 2 (*ARMS2*, also called *LOC387715*) also be related to patients' susceptibility to macular inflammation (Fritsche et al., 2010; Macdonald and Miller, 2011). There is no effective drug or strategy to improve this debilitating visual disease. Therefore, development of novel therapies for AMD is urgently needed.

The pathogenesis of AMD remains unclear, but oxidative stress and the production of reactive oxygen species (ROS) are perhaps responsible for the aging sequence of retina and may play an important role in the pathogenesis of macular degeneration (Blasiak et al., 2014). Oxidative stress can affect both the lipid rich retinal outer segment structure and the light processing in the macula. The response to oxidative stress involves several cellular defense reactions such as increases in antioxidant production and proteolysis of damaged proteins (Winkler et al., 1999). The imbalance between production of damaged cellular components and degradation leads to the accumulation of detrimental products, for example, intracellular lipofuscin and extracellular drusen (Tate et al., 1995). RPE cells are particularly susceptible because of light illumination, high oxygen tension, accumulation of lipid molecules, and other types of stress (Beatty et al., 2000). On the other hand, anti-oxidative enzymes in RPE cells decrease with age, potentially allowing ROS to cause DNA damage and ultimately leading to the apoptosis of RPE cells (Samiec et al., 1998). Although it has been accepted that the RPE is the major pathogenic target of macular degeneration, obtaining a sufficient number of RPE cells from suitable donors for drug screening and disease modeling remains an obstacle.

Human induced pluripotent stem cells (iPSCs) technology had been successfully generated via the retrovirus-mediated transfection of four transcription factors (Oct4, Sox2, Klf-4, and c-Myc) (Takahashi et al., 2007; Yu et al., 2007). Several studies had been made to generate iPSCs from patients with various diseases providing new opportunities for regenerative medicine and *in vitro* disease modeling (Dimos et al., 2008; Park et al., 2008; Maehr et al., 2009). Among disease, those involving retina are necessary and urgent to consider for iPSCs modeling, because of these tissue are not amenable to routine biopsy. In the previous study reported by Osakada et al., human iPSCs were capable of differentiation into differentiated retinal progenitors, RPE cells, and photoreceptors (Osakada et al., 2009). Singh et al. recently also generated Best disease patient-specific iPSCs and differentiated these cells into RPE-like cells and viewed this technique as a great model of degenerative retinal disorder (Singh et al., 2013). Indeed, RPE is one of a few cell types derived from human iPSCs that have met standards for use in human clinical trials (Schwartz et al., 2012). In this study, we isolated T cell from peripheral blood instead of fibroblasts from skin to generate AMD patient-specific induced pluripotent stem cells (AMD-iPSCs). In addition, we used non-integration Episomal vectors instead of integrating viral vectors to reduce the risk of transgenic sequences inserted into the target cell genome. We then differentiated these AMD-iPSCs into RPE-like cells (AMD-RPEs) that were used as an expandable platform for *in vitro* drug screening for investigating the candidate drug that can reduce ROS production and protect RPE cells in AMD.

Curcumin is a natural plant extract from *Curcuma longa L* and widely used in traditional Chinese medicine and in the food industry (Ammon and Wahl, 1991). Curcumin had been demonstrated several types of biological and pharmacological activities, including anti-diabetes, anti-carcinogenic, anti-tumor invasion, anti-angiogenesis activities, anti-oxidant, and anti-inflammation (Aggarwal and Harikumar, 2009; Noorafshan and Ashkani-Esfahani, 2013). In addition, curcumin had been shown to have the possibility of slowing the progress of macular degeneration by protecting RPE cells from light- and oxidant stress- induced cell death and promoting the trafficking of accumulated proteins in retinal cells (Mandal et al., 2009; Woo et al., 2012). In this study, via the AMD patient-specific iPSCs drug screening platform we demonstrated that curcumin serves as an effective scavenger of ROS and enhances the synthesis of antioxidative enzymes in dry AMD iPSC-derived RPE cells. Moreover, pre-treatment of curcumin protected these AMD-related RPE cells from H_2_O_2_-induced cell death. This study suggested that iPSCs were a useful for purposes of personalized medicine. Our results of drug selection using iPSC-derived RPE cells may show us an alternative way to delay oxidative RPE cell death and help prevent the exacerbation of macular degeneration.

## Materials and methods

### T cell activation and expansion

**Figure 1:**
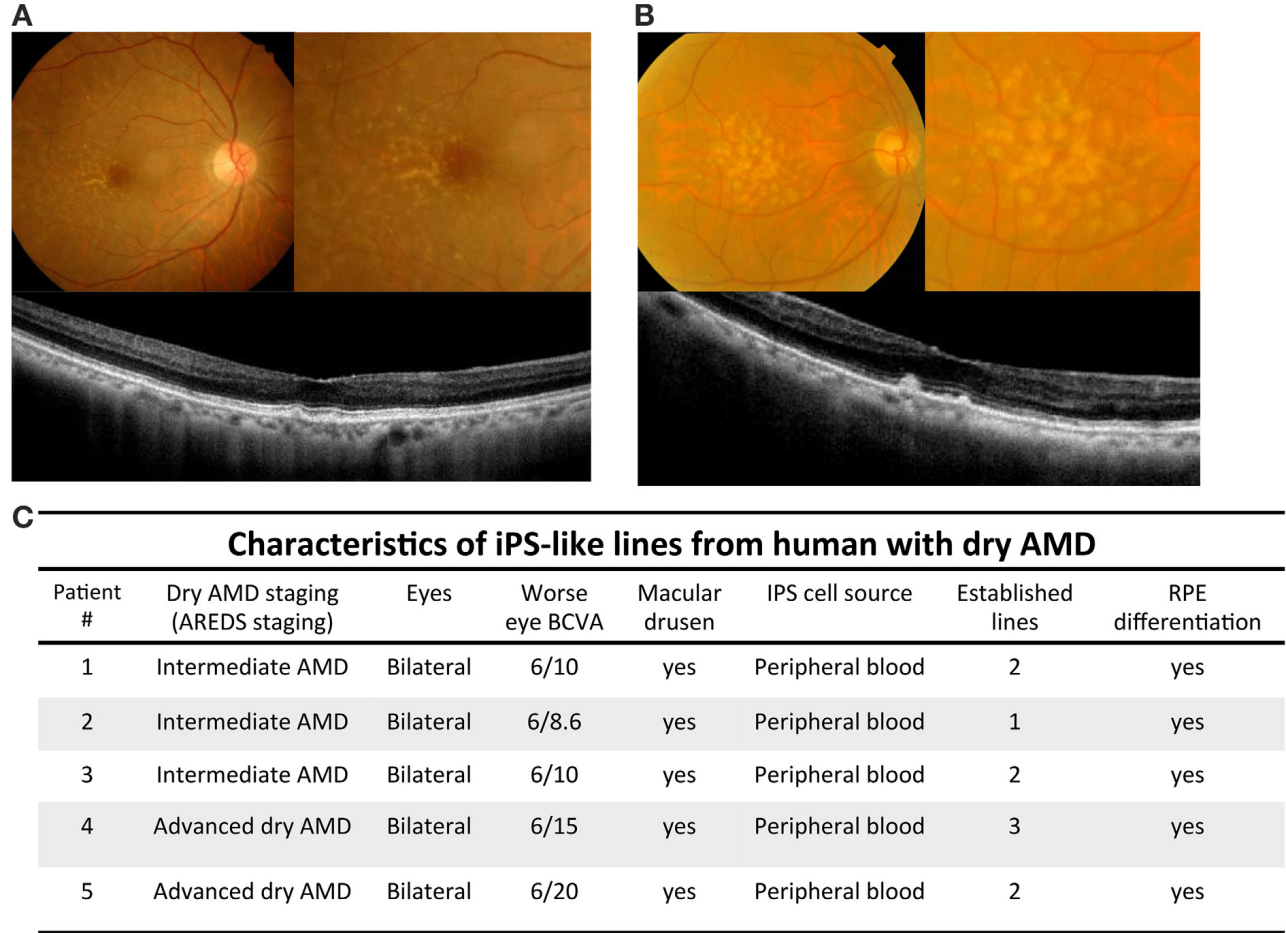
**Imaging findings and characteristics of patients with dry AMD. (A)** Color fundus and optical coherence tomography scans of patient with intermediate dry AMD. Upper left: yellowish depositions, drusen, were detected by color fundi photograph. Upper right: magnified picture of posterior pole revealed drusen locating temperol and adjacent to macular regions. Lower: optical coherence tomography images across the fovea showed small drusenoid depostions. **(B)** Color fundal picture and optical coherence tomography scans of patient with advanced dry AMD. Upper left: Extensive intermediate to large drusen were detected by color fundal photograph. Upper right: magnified pictures of macula revealed extensive drusen all over posterior pole. Lower: optical coherence tomography images across the fovea showed elevated drusenoid depostions with RPE irregularities. **(C)** Demographic information of iPSC cell lines generated from peripheral blood cells of patients with intermediate and advanced dry AMD.

This research followed the tenets of the Declaration of Helsinki, and protocols for this study were approved by the by the Internal Research Board of Taipei Veterans General Hospital (Board No. 2013-11-012B). All samples were obtained after patients had given informed consent. PBMC was isolated from five patients with dry AMD and two unaffected control using Ficoll-Plaque Plus (Amersham Biosciences) according to the manufacturer's protocol. The characteristics of 5 patients with AMD were summarized in Figure 1C. In brief, one ratio of blood sample was layered on one ratio of Ficoll-Plaque Plus, pellet (400 × g, 30 min at 20°C) and the buffy coat was collected, washed twice with PBS and cultured in DMEM (Sigma) (100 IU/mL of penicillin, 100 μ g/mL of streptomycin [Flowlab] and 10% v/v Fetal Bovine Serum [FBS] [PAA, Austria]). T cells were expanded in freshly prepared AIM-V Medium (Invitrogen) supplemented with pen/strep/glutamine (Invitrogen) plus 300 IU/ml rhIL2 (Peprotech) and 10 ng/ml soluble anti-CD3 antibody (eBioscience, OKT3 clone) (Berger et al., 2003).

### Human iPSCs generation and culture

To generate integration-free iPSCs, cells were nucleofected with 3 μg expression plasmid mixture using Amaxa™ human T Cell Nucleofector™ Kit (Lonza). In each nucleofection, 0.83 μg PCXLE-hOCT3/4-shp53, 0.83 μg PCXLE-hSK, 0.83 μg pCXLE-hUL, and 0.5 μg pCXWB-EBNA1 were used. 2 × 10^6^ cells were nucleofected with Amaxa Nucleofector II using program V-024. Cells were cultured the exactly same way as for reprogramming with lentiviral vector expect that every 10–14 days, freshly thawed inactivated mouse embryonic fibroblasts (MEFs) feeder cells were added into each well. The number of ALP-positive iPSC colonies was counted at 3–4 weeks after nucleofection. Undifferentiated iPSCs were maintained on inactivated MEFs (50,000 cells/cm^2^) in human ESC medium (DMEM/F12 (Gibco) supplemented with 20% KnockOut serum replacer (KSR; Invitrogen), 0.1 mM non-essential amino acids (Invitrogen), 1 mM L-glutamine, 0.1 mM ß-mercaptoethanol, 10 ng/ml recombinant human basic fibroblast growth factor (bFGF), and antibiotics (Gibco). To prevent cell contamination by MEFs, these iPSCs were transferred to feeder-free/serum-free culture in HESF V2 medium (Cell Science & Technology) without KSR supplementation as described previously (Chien et al., 2012).

### Differentiation for retinal pigment epithelial cells

The iPSCs were differentiated to RPE according to the protocol established by Dr. Osakada (Osakada et al., 2009). The iPSCs clumps were first incubated in human ES cell culture medium supplemented with 10 μM Y-27632 (WAKO), 5 μM SB431542 (Sigma–Aldrich) and 3 μM CKI-7 (Sigma–Aldrich) for 1 day. The cells were incubated in a differentiation medium (Glasgow minimum essential medium [GMEM; Invitrogen], 1 mM sodium pyruvate, 0.1 mM non-essential amino acids, and 0.1 mM 2-mercaptoethanol) containing 20% knockout serum replacement (KSR; Invitrogen) for 4 days, then in 15% KSR-containing differentiation medium for 6 days, and finally in 10% KSR-containing differentiation medium for 11–40 days. Y-27632 (10 μM), SB431542 (5 μM) and CKI-7 (3 μM) were added to the differentiation medium for the first 13 and 19 days, respectively. Partially differentiated cells were dissociated and incubated on non-adhesive dishes (Corning) in RPE maintenance medium (DMEM:F12 [7:3] supplemented with B-27 supplement [Invitrogen] and 2 mM l-glutamine [Invitrogen]) for 10 days. The resulting RPE cell aggregates were isolated and replated on CELLstart- (Invitrogen) coated dishes in RPE maintenance medium supplemented with 0.5 μM SB431542 and 10 ng/ml bFGF. The medium was changed every 2–3 days. Thereafter, RPE cells formed compact monolayers and re-pigment, typically 90–120 days.

### Alkaline phosphatase assay

The cells were fixed with 4% paraformaldehyde and washed with PBST. The alkaline phosphatase activity was determined by the Alkaline Phosphatase Substrate Kit III (Vector Labs) following the manufacturer's protocol. Colonies stained red indicated positive alkaline phosphatase activity.

### Reverse transcription-polymerase chain reaction (RT-PCR)

Total RNA was isolated with TRIzol Reagent (Invitrogen) and quantified by spectrophotometry at 260 nm. On a GeneAmp® PCR System 9700 thermocycler (Applied Biosystems), 5 μ g of each total RNA was reverse-transcribed with SuperScript III (Invitrogen) at 55°C for 1 h into total complementary DNA, which was then used as the template for the subsequent PCR reactions and analysis. The PCR reactions involved an initial denaturation at 94°C for 5 min, followed by 25 or 30 cycles at 94°C for 30 s, exposure to an appropriate annealing temperature (58–62°C) for 30 s, and then a final incubation at 72°C for 45 s. The primers and cycling conditions for real-time RT-PCR were shown as Table 1. Amplified RT-PCR products were then analyzed on 1% agarose gels and visualized using ethidium bromide staining and a camera system (Transilluminator/SPOT; Diagnostic Instruments). The gel images of the RT–PCR products were directly scanned (ONEDscan 1-D Gel Analysis Software; Scanalytic Inc.), and the relative densities were obtained by determining the ratio of the signal intensity to the GAPDH or β-actin band.

**Table 1:** **The sequences for the primers of RT-PCR**.

**Gene**	**Forward primer**	**Reverse primer**
endo Oct4	GACAGGGGGAGGGGAGGAGCTAGG	CTTCCCTCCACCCAGTTGCCCCAAAC
endo Sox2	GGGAAATGGGAGGGGTGCAAAAGAGG	TTGCGTGAGTGTGGATGGGATTGGTG
endo Klf4	ACGATCGTGGCCCCGGAAAAGGACC	TGATTGTAGTGCTTTCTGGCTGGGCTCC
endo c-Myc	GCGTCCTGGGAAGGGAGATCCGGAGC	TTGAGGGGCATCGTCGCGGGAGGCTG
Nanog	CAGCCCCGATTCTTCCACCAGTCCC	CGGAAGATTCCCAGTCGGGTTCACC
REX	CAGATCCTAAACAGCTCGCAGAAT	GCGTACGCAAATTAAAGTCCAGA
DPPA2	CCGTCCCCGCAATCTCCTTCCATC	ATGATGCCAACATGGCTCCCGGTG
GDF3	CTTATGCTACGTAAAGGAGCTGGG	GTGCCAACCCAGGTCCCGGAAGTT
RLBP1	GCCATGGTACTTCACCACGA	GGCTCCTTGCCTGTTTTCAC
RPE65	ACCCAGTGGGGGAAGATTAC	GCAGCAGAGATCCACAATCA
MITF	ACCATCAGCAACTCCTGTCC	CCAGTTCCGAGGTTGTTGTT
PAX6	GAGGTCAGGCTTCGCTAATG	CTCACACATCCGTTGGACAC
β-Actin	GCATTGCTTTCGTGTAAATTATGT	ACCAAAAGCCTTCATACATCTCA
GAPDH	GTCGCCAGCCGAGCCACATC	CCAGGCGCCCAATACGACCA

### Immunofluorescence staining

The living cells and spheres were fixed in 4% paraformaldehyde, permeabilized in 0.1% Triton X-100, and blocked in 5% normal goat serum-PBS. Cells were incubated with primary antibodies, and the antibody and conditions were shown as Table 2. After being washed three times in PBS, the cells were then incubated with goat anti-mouse or secondary antibodies conjugated with FITC (green) or PE (red). DAPI was used as nuclear stain (blue). Images were obtained using fluorescent microscopy and a digital camera.

**Table 2:** **List of proteins tested by antibodies**.

**Protein**	**Assay**	**Origin**	**Dilution**	**Incubation period**
Oct-3/4	IF	Cell signaling #2840	1:200	4°C Overnight
NANOG	IF	Cell signaling #3580	1:500	4°C Overnight
Tira-1-60	IF	Cell signaling #4746	1:800	4°C Overnight
Tira-1-81	IF	Cell signaling #4745	1:800	4°C Overnight
Nestin	IF	CHEMICON AB5922	1:200	4°C Overnight
AFP	IF	Cell signaling #3903	1:200	4°C Overnight
SMA	IF	Millipore #04-1094	1:200	4°C Overnight
ZO1	IF	Zymed 61-7300	1:200	RT 1 h
RPE65	IF	Abcam ab13826	1:200	RT 1 h
p-JNK	WB	Invitrogen 36-9300	1:2000	4°C Overnight
JNK	WB	Cell signaling #9258	1:2000	4°C Overnight
GAPDH	WB	GeneTex GTX100118	1:10000	4°C Overnight

Abbreviations: WB, Western blot; IF, immunofluorescence; RT, room temperature.

### Quantitative RT-PCR

The total RNA (5 μ g) of each sample was reversely transcribed in 20 mL using 0.5 mg of oligo dT and 200 U Superscript II RT (Invitrogen). The amplification was carried out in a total volume of 20 ml containing 0.5 mM of each primer, 4 mM MgCl2, 2 ml LightCycler FastStart DNA Master SYBR green I (Roche Diagnostics) and 2 ml of 1:10 diluted cDNA. The quantification of the unknown samples was performed by LightCycler Relative Quantification Software, version 3.3 (Roche Diagnostics). PCR reactions were prepared in duplicate and heated to 95°C for 10 min followed by 40 cycles of denaturation at 95°C for 10 s, annealing at 55°C for 5 s, and extension at 72°C for 20 s. All PCR reactions were performed in duplicate. Standard curves (cycle threshold values versus template concentration) were prepared for each target gene and for the endogenous reference (GAPDH) in each sample. The primers for quantitative real-time RT-PCR were shown as Table 3.

**Table 3:** **The sequences for the primers of quantitative real time RT-PCR**.

**Gene**	**Forward primer**	**Reverse primer**
GAPDH	CAGAACATCATCCCTGCCTCTAC	TTGAAGTCAGAGGAGACCACCTG
SOD2	GGGGTGCTGGTTTGCGTCGT	GACCTGCACTGGTACAGCCTGC
GPX1	TGGGCATCAGGAGAACGCCA	GCGTAGGGGCACACCGTCAG
HO-1	TCATGAGGAACTTTCAGAAGGGCC	TCTCCTTGTTGCGCTCAATCTCCT
VEGF	AGCACATAGGAGAGATGAGCTTCC	ACGTCTGCGGATCTTGTACAAAC
FDGF	TGTTCCAGATCTCGCGGAAC	GCGGCCACACCAGGAAG
IGFBP-2	GCGCGGGTACCTGTGAAA	CTACTGCTGGTGAGACTCCCT

### Intracellular reactive oxygen species (ROS) production

The measurement of intracellular ROS production by the probe 20, 70-dichlorofluorescein diacetate (DCFH-DA; Molecular Probes) was mentioned previously (Woo et al., 2012). In brief, cells were incubated with 5 mmol/L DCFH-DA in culture medium for 30 min at 37°C, followed by washing with PBS and flow cytometry analysis.

### MTT assay

For evaluation of cell survival, cells were seeded on 24-well plates at a density of 2 × 10^4^ cells/well, followed by the addition of methyl thiazol tetrazolium (MTT; Sigma) at the end of cell culture. The amount of MTT formazan product was determined using a microplate reader at an absorbance of 560 nm (SpectraMax 250, Molecular Devices).

### Enzyme-linked immunosorbent assay (ELISA)

After 3 days of RPE cells incubation, culture media was collected and measured for PDGF-BB (Abcam; ab100624), VEFG (Abcam; ab100663) and IGFBP-2 (Abcam; ab100540) levels using human ELISA kit)according to the manufacturer's protocol. The optical densities were determined within 30 min and recorded with a microplate reader (BioTek, EXL800, Winooski, VT) at 450 nm.

### Western blot assay

Whole cell lysates were separated by electrophoresis on 12% SDS–PAGE and transferred to polyvinylidene fluoride membrane. The membranes were blocked with 5% nonfat milk at room temperature for 1 h. The primary antibodies in TBST buffer containing 3% nonfat milk at 4°C overnight and subsequently with secondary antibody conjugated with peroxidase at 25°C for 1 h. The immunoblots were developed using an enhanced chemiluminescence system, and the luminescence was visualized on X-ray film. The antibodies for western blot are shown as supplementary Table 2.

### Statistical analysis

The results were expressed as the mean ± SD. Statistical analyses were performed using the *t*-test for comparing 2 groups, and one-way ANOVA was used to detect differences among 3 or more groups. The results were considered statistically significant at *P* < 0.05.

## Results

### Clinical findings of dry type age-related macular degeneration

Numerous studies have focused on human iPSCs as a result of their ability to generate infinitely and maintain the ability to differentiate into various cell types (Takahashi et al., 2007). In this study, we isolated T cell from dry type AMD patients to generate patient-specific iPSCs and then differentiated these iPSCs into RPE-like cells that were used as an expandable platform for *in vitro* drug screening. We obtained 20 ml peripheral blood from the donors (including five patients with dry AMD and two unaffected control). Based on age-related eye disease study (AREDS) staging, we divided these cases into intermediate and advanced dry AMD. Intermediate dry AMD is defined as at least one eye expressing one or more intermediate drusen (63~124 μm in diameter), extensive small drusen, or pigment abnormalities associated with AMD. Advanced dry AMD is defined as at least one eye having one or more large drusen (>125 μm in diameter) or extensive intermediate drusen. Color fundi photographs of intermediate dry AMD showed the presence of numerous small to intermediate drusen, deposited primarily between the temporal and macula regions. Horizontal 6-mm optical coherence tomography scans revealed AMD retinas with drusenoid depositions in the macular area (Figure 1A). In the cases with advanced dry AMD, color fundi pictures revealed extensive intermediate to large drusen in macula and the posterior pole. Horizontal 6-mm optical coherence tomography scans revealed AMD retinas with macular depositions and RPE irregularities (Figure 1B). The demographic data in these 5 cases are summarized in Figure 1C. The patients were aged from 69–86-years-old. iPS cell lines were achieved successfully in all 5 cases with subsequent RPE differentiation.

### Generation of iPSCs and RPEs from AMD patients

**Figure 2:**
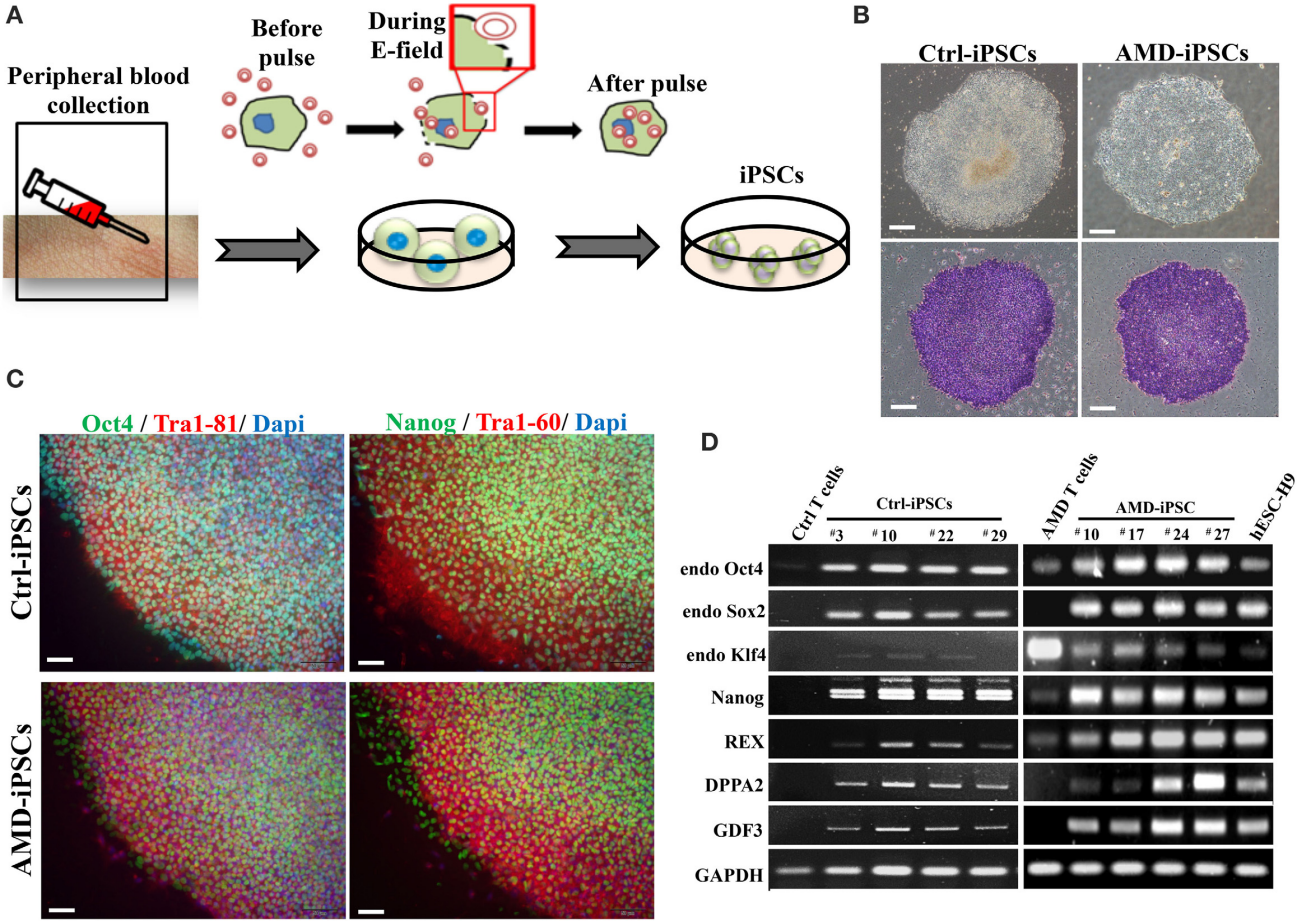
**Generation and characterization of iPSCs derived from patients with AMD and unaffected control. (A)** Schematic diagram of the reprogramming with episomal plasmids via electroporation. **(B)** Phase-contrast photomicrograph and alkaline phosphatase activity of undifferentiated control iPSCs (Ctrl-iPSCs) and AMD iPSCs (AMD-iPSCs). Scale bar = 100 μm. **(C)** Immunofluorescence staining demonstrated the expression of pluripotency markers (OCT4, TRA-1-61, NANOG, and TRA-1-81) in undifferentiated iPSCs from patients and unaffected. Nuclei were counterstained with DAPI (blue). Scale bar = 30 μm. **(D)** The RT–PCR results indicated an ESC-like gene expression pattern in representative colonies of iPSCs. T cells were used as a negative control, and human ES H9 cells were used as a positive control.

**Figure 3:**
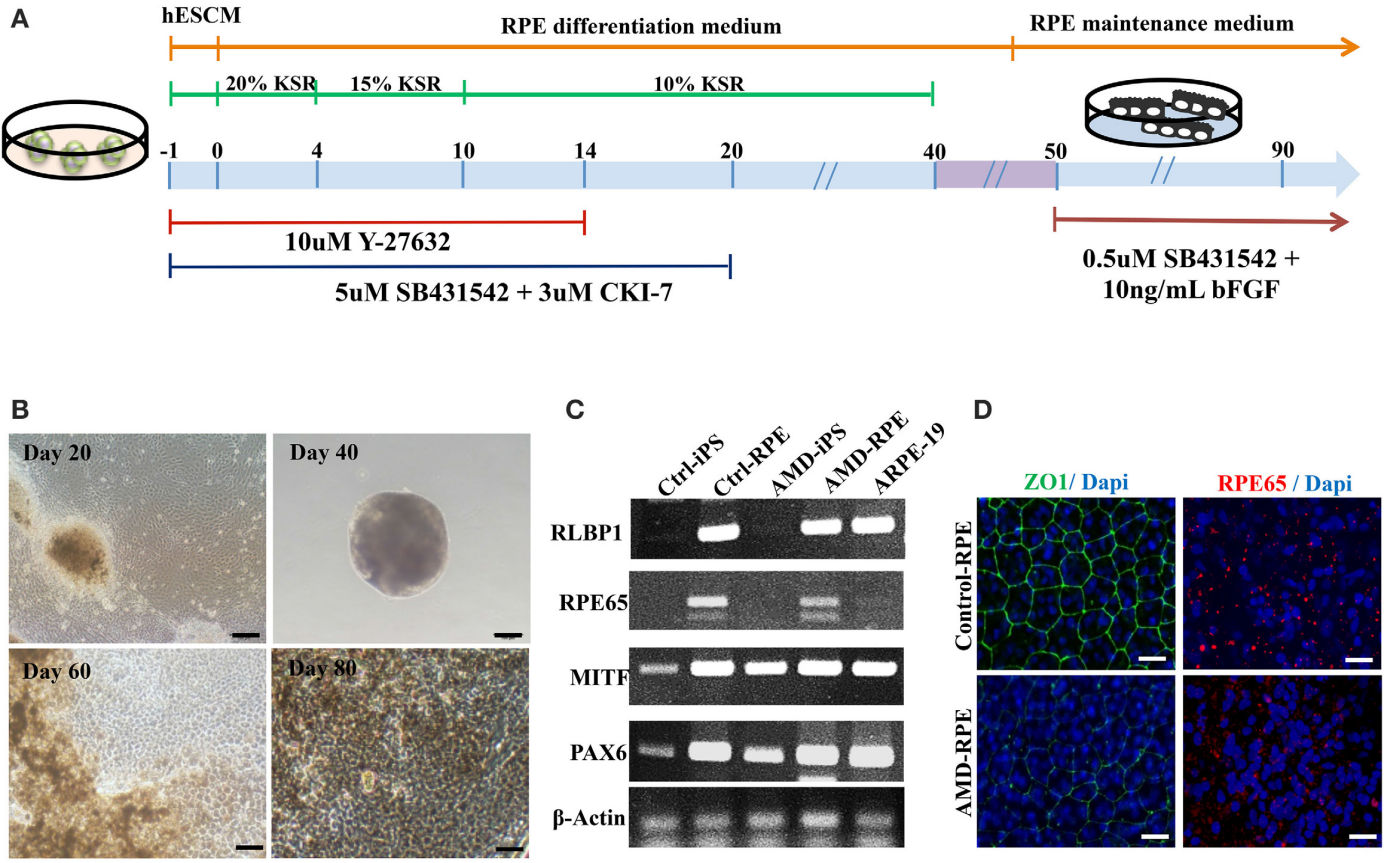
**Differentiation of induced pluripotent stem cells into retinal pigment epithelial cells. (A)** Schematic diagram of the culture procedure for RPE differentiation. **(B)** Light microscopic images of RPE cells derived from control and AMD iPSCs confirmed characteristic RPE morphology and pigmentation. Scale bars = 20 μm. **(C)** RT–PCR analysis revealed the expression of characteristic RPE genes. **(D)** Immunofluorescence staining for ZO-1 and RPE65 demonstrated morphology and tight junction formation in Ctrl-RPEs compared with AMD-RPEs.

AMD results from RPE dysfunction or loss associated with photoreceptor fallout, Bruch's membrane thickening, and choriocapillary hypoperfusion (Young, 1987). However, obtaining a number of suitable RPE cells for *in vitro* study is still a problem. Although fibroblasts from skin biopsy or other sources (such as dental pulp cells) were used in many studies for the generation of iPSCs, peripheral blood mononuclear cells (PBMCs) have been widely accepted as a more convenient and an almost unlimited resource for cell reprogramming (Staerk et al., 2010; Seki et al., 2012). In this study, we used EBNA1-based episomal vectors, a non-viral system that can reprogram somatic cells into iPSCs in both feeder-dependent and feeder-free conditions, to generated iPSCs from T cells via electroporation (Figure 2A). These integration-free iPSCs have the capability to be utilized for a broad range of applications, including pre-clinical research and human gene therapy, thus further delivering on the promise of iPSCs. These reprogrammed cells progressively formed colonies with increasing size during the reprogramming process. These colonies were stained positive for alkaline phosphate (ALP), and exhibited morphology indistinguishable from that of human ESCs (Figure 2B). As shown by immunofluorescence, selected clones exhibited the stamens signature and revealed the strong expression of Oct4, Nanog, Tra1-60, and Tra1-81 in the 20th-passage iPSCs (Figure 2C). RT-PCR also showed that both Ctrl- and AMD-iPSCs expressed various stemness genes, such as Oct4, Sox2, klf4, Nanog, REX, DPPA2, and GDF3, identical to those observed in H9 human ESC lines (Figure 2D). Using differentiation protocols for tridermal lineages, both the 20th-passage Ctrl-iPSC-derived embryoid bodies (EBs) and AMD-iPSC-derived EBs could be induced to differentiate into neuron-like cells, smooth muscle cells, and hepatocyte-like cells (ectoderm, mesoderm, and endoderm) (data not shown). These results demonstrated that AMD patient-derived iPSCs exhibited pluripotent properties and capabilities of multi-lineage differentiation. We further employed the culture protocol described by Osakada et al., with brief modifications, for RPE differentiation (Osakada et al., 2009). We used patient-specific iPSC-derived RPE cells to model the pathophysiological features of macular degeneration (Figure 3A). Using this protocol, both Ctrl- and AMD-iPSCs underwent RPE-specific morphological changes and pigmentation and differentiated into RPE-like cells (Figure 3B). RT-PCR confirmed the expression of several RPE-specific markers, including RLBP1, RPE65, MITF, and PAX6, in both Ctrl-RPEs and BD-RPEs (Figure 3C). Immunofluorescence assays indicated that AMD-RPEs exhibited significantly lower ZO-1 and RPE65 expression than Ctrl-RPEs, suggesting tight-junction in AMD-RPEs were defective (Figure 3D). These data demonstrated that both Ctrl- and AMD-iPSCs were competent to differentiate into pigment cells with typical RPE characteristics.

### iPSC-derived RPEs as a drug screening platform of macular degeneration

**Figure 4:**
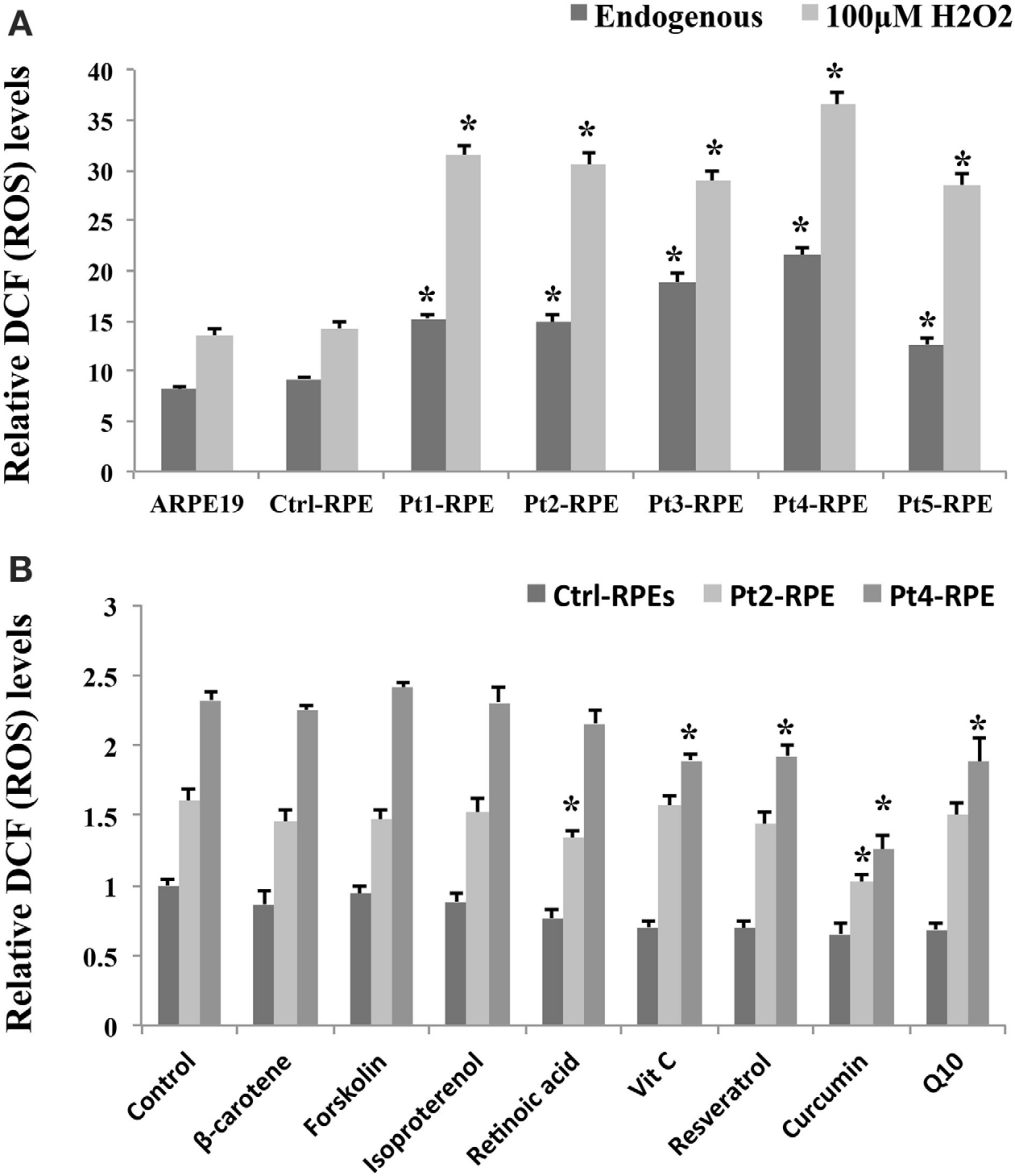
**Accumulation of endogenous ROS in AMD-RPEs as a drug screening platform. (A)** Endogenous and H_2_O_2_-induced ROS were measured with flow cytometry. In H_2_O_2_-induced ROS group, RPE cells were separately pretreated with 200 μ M H_2_O_2_ for 6 h. Each bar represents the mean ± SD of three independent experiments (^*^p < 0.05, compared with ctrl-RPE). **(B)** Endogenous ROS were measured by flow cytometry after treatment with dietary supplements for retinal protection and natural compounds for anti-oxidative stress for 24 h (10 μ M β-carotene, 10 μ M retinoic acid, 5 μ M forskolin, 200 μ M isoproterenol, 10 μ M resveratrol, 10 μ M vitamin C, 10 μ M curcumin, and 20 μ M Q10). Each bar represents the mean ± SD of three independent experiments (^*^p < 0.05, compared with non-treated control).

The RPE is a monolayer of pigmented cells forming part of the blood retina barrier and is particularly susceptible to oxidative stress because of the layer's high consumption of oxygen. Thus, chronic oxidative stress induces RPE damage that is responsible for the aging process and plays a significant role in the pathogenesis of AMD (Beatty et al., 2000; Yildirim et al., 2011). Our data revealed a higher accumulation of endogenous ROS in AMD-RPEs than Ctrl-RPEs as well as a more significantly increased ROS production when additional treatment by H_2_O_2_ (Figure 4A). Several dietary supplements for retinal protection and natural compounds for anti-oxidant, including β-carotene, lutein, retinoic acid, forskolin, isoproterenol, resveratrol, vitamin C, curcumin, and Q10, were selected in the present drug screening study. To determine the safe dosage for each selected candidate drug, the cell viability of AMD-RPEs after 24 h incubation with the selected drugs at various concentrations was measured by MTT assay (data not show). We selected the highest dosage that did not affect cell viability (with no cytotoxic or proliferative effects) as the optimal treatment dose of each drug (i.e., forskolin and lutein: 5 μ M; β-carotene, retinoic acid, resveratrol, vitamin C, and curcumin: 10 μ M; Q10: 20 μ M; isoproterenol: 200 μ M). Among all of the above-mentioned candidate drugs, curcumin reduces the ROS production to a large extent in AMD (Figure 4B). Based on these findings in the drug screening study, we speculated that curcumin could be an effective drug with therapeutic effects against oxidative stress in macular degeneration.

### Effect of curcumin on H_2_O_2_-induced cell death and ROS generation

**Figure 5:**
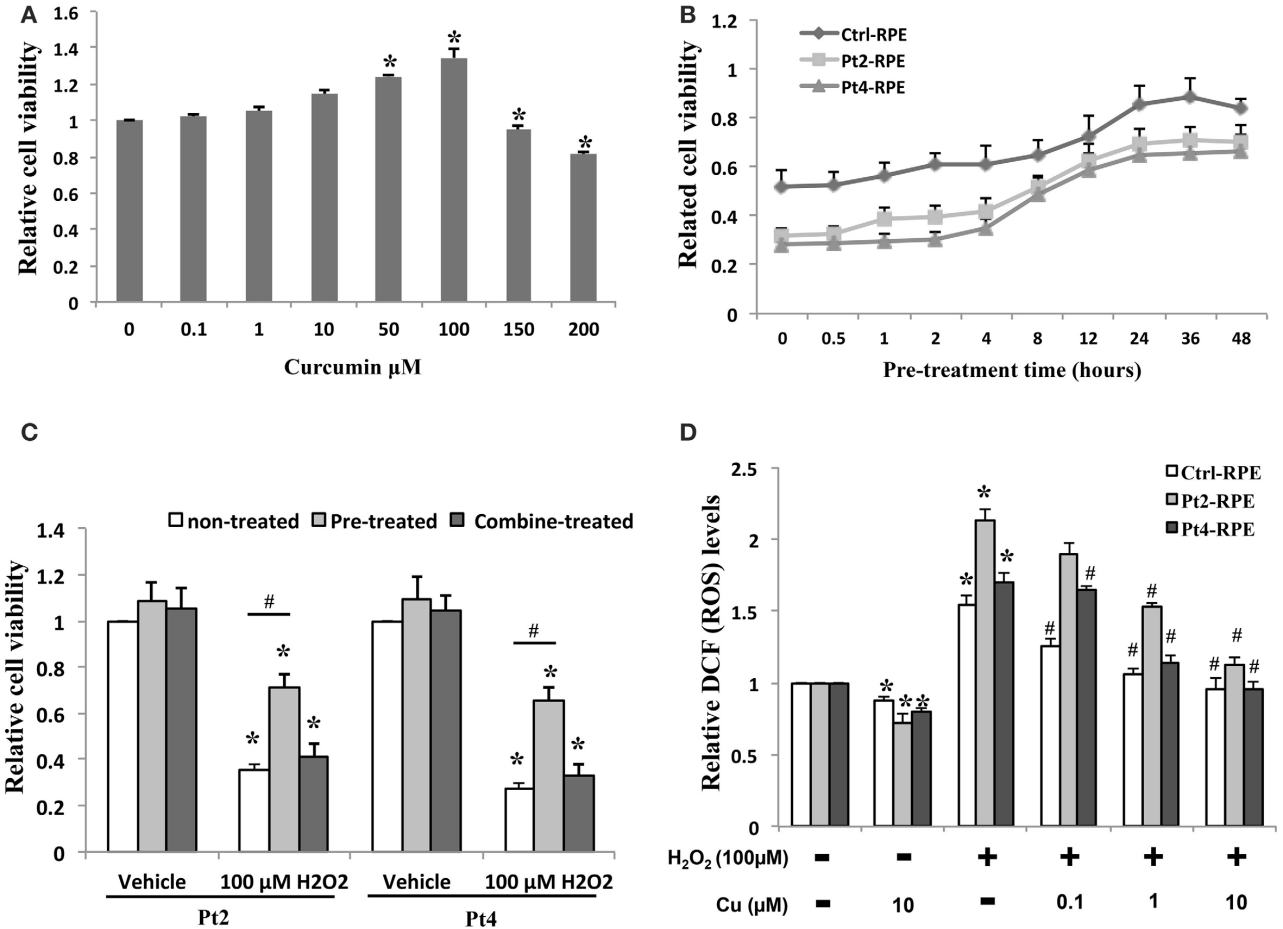
**Protective effect of curcumin on AMD-RPEs. (A)** Cell viability in AMD-RPEs treatment with different concentrations of curcumin for 24 h. Each bar represents the mean ± SD of three independent experiments (^*^p < 0.05, compared with non-treated control). **(B)** Time effect of curcumin on cells pretreated with 10 μ M curcumin at different time periods prior to 200 μ M H_2_O_2_ exposure for 6 h. Each bar represents the mean ± SD of three independent experiments. **(C)** Cell viability was measured in AMD-RPEs combine or 12 h pretreated with10 μ M curcumin or treated with 10 μ M curcumin plus 200 μ M H_2_O_2_ for 6 h. Each bar represents the mean ± SD of three independent experiments (^*^p < 0.05, compared with vehicle control; ^#^*p* < 0.05. compared with non-treated control). **(D)** ROS production was measured with flow cytometry for indicated conditions. RPEs were pretreated with curcumin for 12 h and plus 100 μ M H_2_O_2_ for 6 h. Each bar represents the mean ± SD of three independent experiments (^*^p < 0.05, compared with non-curcumin and non-H_2_O_2_ treated control; #*p* < 0.05, compared with non-curcumin but 100 μ M H_2_O_2_ treated group).

Ctrl- and AMD-RPEs were treated with curcumin at 0.1–200 μ M for 24 h, and cell viability was determined by MTT assay. The non-treated and lower concentrations curcumin-treated groups (0.1, 1, and 10 μM) showed no significant difference in cell viability. We also found that curcumin at concentrations above 10 μM increased the proliferation of RPE cells. At higher concentrations (150 and 200 μM), curcumin decreased the viability of the cells (Figure 5A). To determine the protective effects of curcumin on H_2_O_2_-induced cell death, ctrl- and AMD-RPEs were pretreated with 10 μ M curcumin at different time periods (0, 0.5, 1, 2, 4, 8, 12, 24, 36, and 48 h) prior to 200 μ M H_2_O_2_ exposure for 6 h. MTT assay demonstrated that curcumin exhibited the protective effect against H_2_O_2_-induced cell death when its pretreatment time was less than 8~12 h (Figure 5B). To exclude a direct protective effect of curcumin, the cells were combined treatment with 10 μ M plus 200 μ M H_2_O_2_ for 6 h. Our results showed that the cell viability was not significantly different as compared to the treatment with H_2_O_2_ alone, suggesting that curcumin had no direct protective effects on the high dose H_2_O_2_-induced cell death (Figure 5C). To evaluate the inhibitory effect of curcumin on ROS production in ctrl- and AMD-RPE cells, we investigated whether curcumin could inhibit H_2_O_2_-induced ROS production. AMD- RPEs were pretreated with different concentrations (0.1, 1, and10 μ M) of curcumin for 12 h prior to 100 μ M H_2_O_2_ exposure for 6 h. As shown in Figure 5D, intracellular ROS was significantly increased by H_2_O_2_. Administration of curcumin decreased and maintained intracellular ROS at low levels in AMD-RPEs. These data suggest that curcumin effectively prevented AMD-RPEs from H_2_O_2_-induced cell death and inhibited ROS production in these RPE cells.

### Amelioration of oxidative stress in the AMD RPEs by curcumin

**Figure 6:**
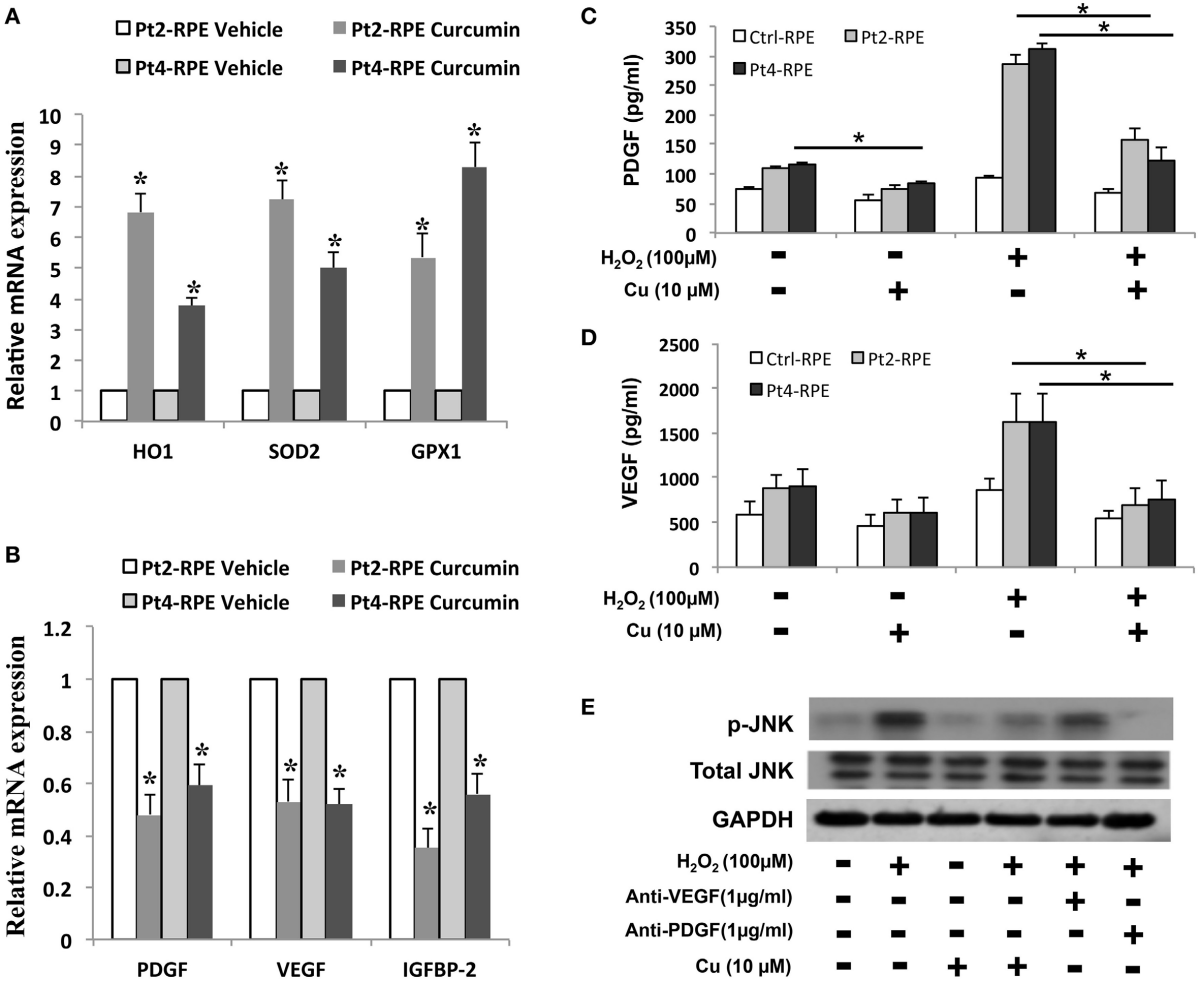
**Modulation of curcumin in oxidative stress-regulating genes**. qRT-PCR was used to quantify the relative amounts of **(A)** antioxidant genes and **(B)** growth factors in the indicated groups. Each bar represents the mean ± SD of three independent experiments (^*^p < 0.01 compare with vehicle control). Levels of platelet-derived growth factor (PDGF) and **(C)** vascular endothelial growth factor (VEGF) **(D)** of AMD-RPEs in the absence and presence of H_2_O_2_-stimulated and/or pre-treatment of curcumin. Culture medium was assayed for enzyme-linked immunosorbent assay (ELISA) as described in the methods section (^*^p < 0.05). Data represents three independent experiment and all data points plotted as mean ± SD (^*^p < 0.05). **(E)** Representative blots show comparative protein levels of total and phosphor- JNK-1/2 in the AMD-RPEs.

Because the reduction of oxidative stress has been proposed to be protecting against macular degeneration, we further tested effects of therapeutic agents of curcumin, a naturally-occurring compound with known anti-inflammatory and anti-oxidative properties, in AMD iPSC-derived RPE. Curcumin significantly attenuated this increase, indicating that curcumin acts as an ROS scavenger in this system (Figure 5D). To understand the molecular mechanism of curcumin-mediated protection of RPEs against oxidative stress, we performed quantitative RT-PCR to measure the expression of genes known to be involved in cellular stress and inflammatory processes. AMD-RPEs exhibited generally lower levels of antioxidant genes including HO-1, SOD2 and GPX1 when comparing with Ctrl-RPEs. After administration of curcumin, the expression of these antioxidant genes was significantly up-regulated (Figure 6A). Recent evidences have demonstrated that curcumin may modulate growth factor expression and can act as a therapeutic agent (Epstein et al., 2010; Hollborn et al., 2013). PDGF, VEGF, and IGFBP-2 have been shown to be involved in regular RPE functions or retinal diseases (Epstein et al., 2010; Hollborn et al., 2013). We therefore wanted to determine whether curcumin also regulated the expression of PDGF, VEGF, and IGFBP-2. Quantitative RT-PCR showed that AMD-RPEs express a higher level of these three factors when compare with control, but supplementation of curcumin significantly reduced their expression (Figure 6B). In the next step, we sought to determine whether H_2_O_2_ could change the secretion of PDGF, VEGF, and IGFBP-2. To this end, we compared the concentration of these three secretory proteins in the supernatant of culture medium. Our results showed that the concentration of soluble PDGF and VEGF were obviously increased after treatment of H_2_O_2_ and significantly reduced by pretreated with curcumin (Figures 6C,D). These data suggested that curcumin might inhibit the H_2_O_2_-induced secretion of PDGF and VEGF. It has been demonstrated that the JNK signaling pathway is important in cell death. Thus, we determined the activity of the JNK signaling pathway in H_2_O_2_-stimulated AMD-RPEs with or without the pre-treatment of curcumin. As shown in Figure 6E, western blotting data demonstrated that the phospho-JNK1/2 protein levels in the H_2_O_2_-stimulated AMD-RPEs pre-treated with curcumin were significantly lower than those in the H_2_O_2_-stimulated AMD-RPEs without curcumin pre-treatment, and the total form JNK was not affected. Notably, adding PDGF-neutralizing antibodies to medium consistently abrogated the phosphorylation of JNK in H_2_O_2_-stimulated AMD-RPEs. Taken together, our data suggested the pre-treatment with curcumin could not only significantly increase oxidative stress defense enzyme but also modulate secretion of PDGF and activity of JNK pathway leading to inflammatory processes and cell death in these oxidative-stress-damaged RPEs.

## Discussion

AMD is generally afflicts people over the age of 60-years-old, and is the leading cause of vision loss among the elderly (Jager et al., 2008; Eramudugolla et al., 2013). Risk factors of AMD are various and include aging and different genetic predispositions, together with several environmental/epigenetic factors, such as, cigarette smoking, dietary habits, and phototoxic exposure (Nowak, 2006; Shahid et al., 2012; Stanton and Wright, 2014). To date, there is no specific therapy for curing AMD or improving the visual functional on this progressive disease. No effective agents could be screened until now due to a lack of knowledge and materials about the disease pathogenesis of AMD. This makes it necessary to create study models for studying AMD pathogenesis and to design new therapeutic approaches. Recently, patient-specific iPSCs and their differentiated progenies have provided models for particular individualized disease phenotypes that can be used to understand disease mechanisms and investigate the pathogenesis of disease-causing mutations (Singh et al., 2013; Romano et al., 2014). In addition, the high consistency, purity, and expandability of human iPSCs facilitate drug screening, toxicity testing, and the development of personalized medicine for the treatment of degenerative diseases (Saha and Jaenisch, 2009). Nevertheless, iPSCs technology is complicated because of genome-integrating viral vectors may produce insertional mutations, or even result in increasing the risk of tumor formation due to reactivation of the c-Myc oncogene (Stadtfeld et al., 2008; Eggenschwiler and Cantz, 2009). Recently, the development of non-viral, non-integrating, plasmid-based reprogramming systems has gained popularity as an alternative to traditional retroviral-based reprogramming of cells (Fusaki et al., 2009). In this study, we generated patient-specific iPSC via integration-free Episomal vectors iPSCs instead of integrating viral vectors. The episomal vectors based on the Epstein-Barr Nuclear Antigen-1 (*EBNA-1*) have been proven to generate iPSCs very efficiently without the risk of transgenic sequences inserted into the target cell genome. This method represents one step closer to the clinical application of iPSC-based therapy of macular degeneration. Fibroblasts were commonly used in many studies for the generation of iPSCs, it still suboptimal for large-scale clinical derivation because of the need for invasive skin biopsies and the cost long time for establishing stable cell lines from primary tissue (Su et al., 2013). Reciprocally, mononuclear cells from peripheral blood have been widely accepted as a more convenient and almost unlimited resource for cell reprogramming due to the ease of obtaining patient samples. Additionally, large numbers of frozen blood samples, from living and deceased donors, are stored in biorepositories worldwide (Haase et al., 2009; Loh et al., 2009). We successfully isolated sufficient T cells from only 20 ml peripheral blood of the donors and reprogrammed these T cells into iPSCs. These iPSCs could be stably passaged to 50 passages and retained their pluripotency and ability for tridermal differentiation. Subsequently, we differentiated these iPSCs into RPEs and demonstrated that these RPEs recapitulated multiple pathophysiological features of macular degeneration. These features enabled the use of these patient-specific RPEs as a high-throughput and expandable platform for *in vitro* drug screening.

**Figure 7:**
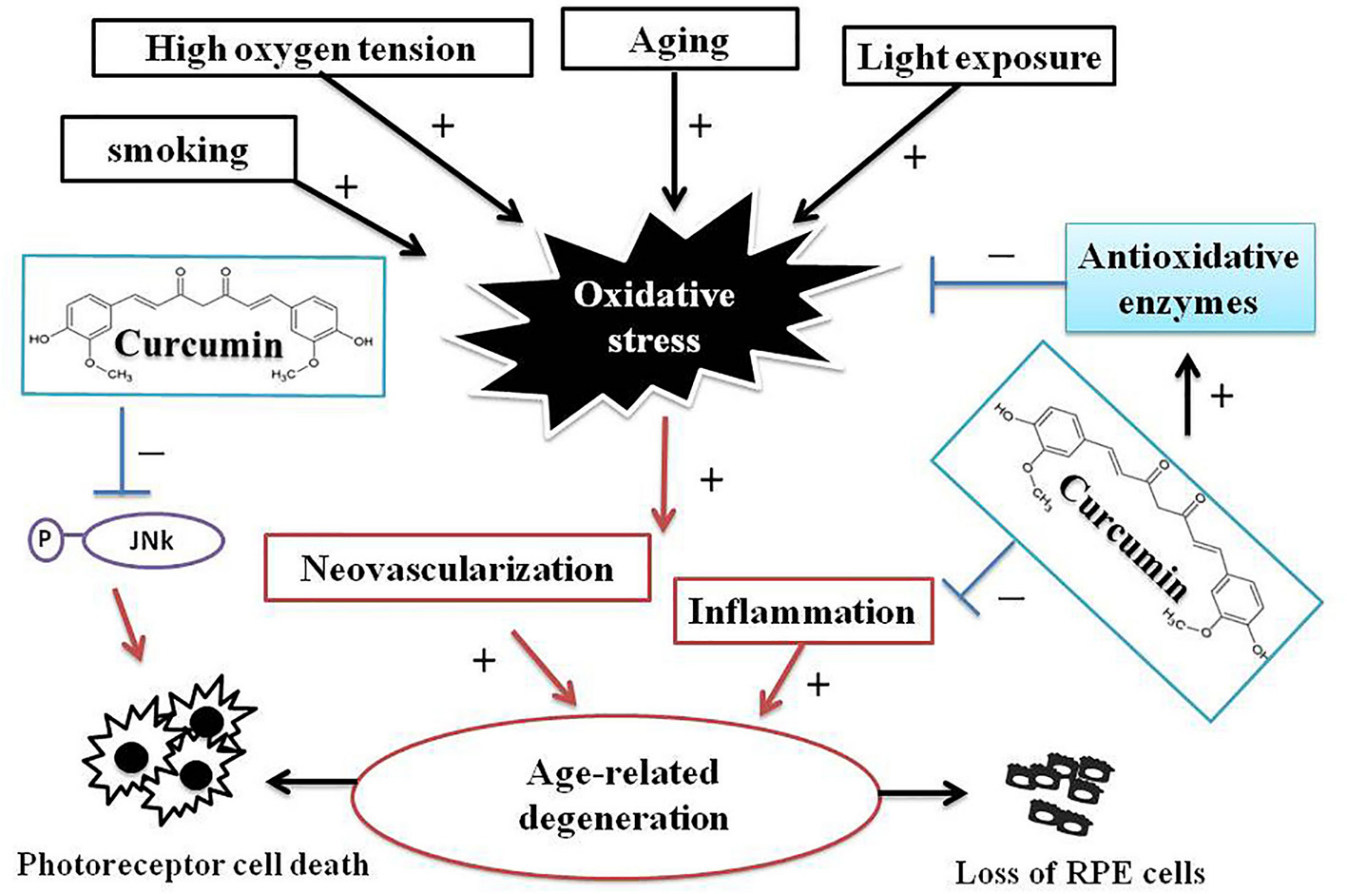
**A schematic illustrations of interrelations between curcumin and processes of AMD pathogenesis**. AMD is a complex chronic neurodegenerative disease associated with many environmental, lifestyle, aging, and genetic factors. Oxidative stress and ROS production play a pivotal role in AMD pathogenesis. Curcumin has remarkable efficacy in ameliorating neurodegenerative, protecting AMD-RPEs from H_2_O_2_-induced ROS and cell death, and the modulating several oxidative stress-regulating genes that may inhibit cellular inflammatory responses and protects AMD-RPEs against oxidative stress. These evidences indicated curcumin may be potentially effective therapeutic means to treat AMD.

The generation of ROS has been considered to have harmful consequences, and has been thought to be a major factor in aging and disease (Handa, 2012). RPE cells are particularly susceptible to oxidative stress, high oxygen tension, lifelong light illumination, and phagocytosis. Therefore, in accordance with the decrease of antioxidative enzymes in RPE cells with age, oxidative stress is thought to play a critical role in the pathogenesis of macular degeneration (Beatty et al., 2000). There is no effective drug or strategy to improve AMD debilitating visual disease. Therefore, the elucidation of its underlying pathogenesis and the development of novel therapies for macular degeneration are urgently needed. Curcumin is widely used in traditional Chinese medicine because of its multifaceted beneficial effects, including anticancer, antioxidant, and neuroprotective properties (Anand et al., 2007; Aggarwal and Harikumar, 2009). In addition, curcumin has shown remarkable efficacy in ameliorating neurodegenerative disorders (Aggarwal and Harikumar, 2009; Jiang et al., 2013) and RPE cell death induced by light or oxidative insults (Mandal et al., 2009; Woo et al., 2012). The efficacy of curcumin has been attributed to the upregulation of oxidative stress defense enzymes (Woo et al., 2012) and the activation of several cellular regulatory proteins that inhibit cellular inflammatory responses and protection (Sreejayan and Rao, 1997; Mandal et al., 2009; Woo et al., 2012). Among several dietary supplements for retinal protection and natural antioxidant compounds, we identified curcumin as a potent agent that can prevent AMD-RPEs from H_2_O_2_-induced cell death and ROS production. In addition, inflammation and angiogenesis have been implicated in the pathogenesis of AMD. Production of cytokines and ROS leads to further RPE and photoreceptor damage. Based on our data, curcumin with its pleiotropic activities can modulate the expression of many oxidative stress regulatory proteins such as PDGF, VEGF, IGFBP-2, HO1, SOD2, and GPX1, which in turn inhibit cellular inflammatory responses and protect RPE cells. Although the mechanisms of curcumin that improved RPE functions in AMD-RPEs were not fully elucidated, our data indicated that, at least in part, a potent ROS scavenging effect was involved in this curcumin-mediated cytoprotection (Figure 7). The *in vivo* therapeutic potential of curcumin for the treatment of AMD should be examined in animal models in future studies.

## Conclusions

This report has demonstrated that patient-specific iPSCs and iPSC-derived RPE-like cells are promising *in vitro* disease models for drug screening. In addition, curcumin may be an ideal drug to effectively reduce ROS production, inhibit H_2_O_2_-induced cell death and impair RPE functions. This intervention strategy represents an ideal model in combination of iPSC-based personalized medicine and nanomedicine technologies. To sum, our work shows that patient-specific iPSCs can act as an efficient platform for drug screening and could help move personalized medicine forward.

### Conflict of interest statement

The authors declare that the research was conducted in the absence of any commercial or financial relationships that could be construed as a potential conflict of interest.
